# Substrate-Limited and -Unlimited Coastal Microbial Communities Show Different Metabolic Responses with Regard to Temperature

**DOI:** 10.3389/fmicb.2017.02270

**Published:** 2017-11-23

**Authors:** Helmut Maske, Ramón Cajal-Medrano, Josué Villegas-Mendoza

**Affiliations:** ^1^Departamento de Oceanografía Biológica, Centro de Investigación Científica y de Educación Superior de Ensenada (CICESE), Ensenada, Mexico; ^2^Facultad de Ciencias Marinas, Universidad Autónoma de Baja California (UABC), Ensenada, Mexico

**Keywords:** marine organotrophic microbes, temperature, growth, respiration, growth efficiency

## Abstract

Bacteria are the principal consumers of dissolved organic carbon (DOC) in the ocean and predation of bacteria makes organic carbon available to higher trophic levels. The efficiency with which bacteria convert the consumed carbon (*C*) into biomass (i.e., carbon growth efficiency, *Y*) determines their ecological as well as biogeochemical role in marine ecosystems. Yet, it is still unclear how changes in temperature will affect *Y* and, hence, the transfer of consumed *C* to higher trophic levels. Here, we experimentally investigated the effect of temperature on metabolic functions of coastal microbial communities inoculated in both nutrient-limited chemostats and nutrient–unlimited turbidostats. We inoculated chemostats and turbidostats with coastal microbial communities into seawater culture medium augmented with 20 and 100 μmol L^−1^ of glucose respectively and measured CO_2_ production, carbon biomass and cell abundance. Chemostats were cultured between 14 and 26°C and specific growth rates (μ) between 0.05 and 6.0 day^−1^, turbidostats were cultured between 10 and 26°C with specific growth rates ranging from 28 to 62 day^−1^. In chemostats under substrate limitation, which is common in the ocean, the specific respiration rate (*r*, day^−1^) showed no trend with temperature and was roughly proportional to μ, implying that carbon growth efficiency (*Y*) displayed no tendency with temperature. The response was very different in turbidostats under temperature-limited, nutrient-repleted growth, here μ increased with temperature but *r* decreased resulting in an increase of *Y* with temperature (*Q*_10_: 2.6). Comparison of our results with data from the literature on the respiration rate and cell weight of monospecific bacteria indicates that in general the literature data behaved similar to chemostat data, showing no trend in specific respiration with temperature. We conclude that respiration rates of nutrient-limited bacteria measured at a certain temperature cannot be adjusted to different temperatures with a temperature response function similar to *Q*_10_ or Arrhenius. However, the cellular respiration rate and carbon demand rate (both: mol C cell^−1^ day^−1^) show statistically significant relations with cellular carbon content (mol C cell^−1^) in chemostats, turbidostats, and the literature data.

## Introduction

Global temperature in the ocean surface layer is expected to increase and this has renewed interest in the metabolic responses (such as growth rate) of unicellular organisms to temperature (Gruber, 2011; Boyd et al., 2013). The classical approach is to characterize the maximum growth rate unlimited by nutrients at a temperature ranging between the cardinal temperatures that delimit the physiological temperature range of microbes defined here as bacteria and archaea. The temperature response is typically interpreted in the framework of the Arrhenius equation by quantifying an activation energy related to the slope of the natural log of the physiological activity over the inverse of the temperature given in Kelvin (Neidhardt et al., 1990). Yet, while the Arrhenius equation has presented itself as a convenient way describing temperature responses of single species populations, it depicts a challenge for communities comprised of many interacting species. For phototroph plankton communities, Eppley (1972) suggested the use of the growth rate vs. temperature space. This space was delimited by the envelope formed by the sum of the maximum growth rates of different phytoplankton species at optimal temperatures; the envelope thus defined maximum growth rates (μ_max_) as a function of temperature but unrestricted by light or nutrients. However, the concept which interprets the metabolic activity of oceanic organotrophic microbes only as a function of temperature is generally not applicable because typically these microbes are substrate limited communities. The interpretation is further complicated because aquatic microbial communities are organized as functional consortia with the exchange of metabolites between species. The knowledge of the species-specific metabolic potential of each member of a consortium would not allow to model its maximum community growth rate because of the exchange of metabolites between members. The metabolic potential of aquatic microbial communities in dependence of temperature can be investigated using nutrient replete continuous cultures, namely turbidostats (Pirt, 1975). The more typical oceanographic condition of microbial communities is substrate limitation which can be investigated using chemostat cultures (Pirt, 1975). In chemostats the nutrient limited microbial community will grow at a rate determined by the nutrient supply rate at a given temperature. Although the growth rate might not change at different temperatures, the temperature could still control the ratio of anabolism to catabolism of the community or of one taxon, resulting in concomitant adjustment of the taxonomic composition of the community and its physiological profile. Thus, the microbial community may change its phenotype and genotype in response to temperature even at a constant nutrient supply rate. According to the classical chemostat concept we would expect the rapid selection of the best adapted taxon for a given temperature and nutrient supply rate, but the close metabolic interaction of microbes within a community supports coexistence in a homogenous environment over time periods that are ecologically relevant.

In the ocean, microbes contribute about half of the upper mixed layer respiration (Carlson et al., 2007; Robinson, 2008) and therefore their rate of respiration in the ocean impacts significantly the metabolic balance between primary production in the ocean and community respiration. The role of bacterial respiration in the ocean carbon flux is still uncertain and has been subject of debate for some time (del Giorgio and Duarte, 2002; del Giorgio et al., 2005). Microbial respiration rates in the ocean are closely linked to the question whether organotrophic microbes are a net sink of organics with low growth rate relative to respiration, implying significant consumption of dissolved organic carbon in relation to biomass produced and little carbon sequestration, a condition defined by low growth efficiency. Alternatively, high growth efficiency would mean a relatively low respiration rate compared to growth rate, which in ecological terms would allow the organotrophic microbes to more effectively feed the higher trophic chain. Currently we lack information on how a temperature increase would affect the growth efficiency of ocean microbes and consequently the carbon cycle in the ocean. The role of microbes can be parameterized by the carbon growth efficiency of microbes (*Y*) calculated from their measured respiration rate (*C*_r_ day^−1^) and microbial production (*C*_b_ day^−1^). *C*_r_ is the CO_2_ mol L^−1^ produced by respiration and *C*_b_ is the mol L^−1^ of microbial carbon biomass produced, See Table 1 for symbols.

(1) $$Y=\left(C_{\text{b}}{\text{day}}^{-1}\right){\left(C_{\text{r}}{\text{day}}^{-1}+C_{\text{b}}{\text{day}}^{-1}\right)}^{-1}$$

**Table 1:** Units and Abbreviations.

**Symbol**	**Explanation**	**Units**
*C*_r_	CO_2_ respired	mol C L^−1^
*C*_b_	Prokaryote carbon biomass	mol C L^−1^
*X*	Cell concentration	cells L^−1^
*M*	Cellular carbon per cell: *C*_b_ *X*^−1^	mol C cell^−1^
*D*	Specific dilution rate	day^−1^
*r*	Specific respiration rate: Δ*C*_r_ $C_{\text{b}}^{-1}$ *t*^−1^	day^−1^
μ	Specific growth rate: Δ*C*_b_ $C_{\text{b}}^{-1}$ *t*^−1^	day^−1^
ρ	Respiration rate per cell: *r M*	mol C cell^−1^ day^−1^
*q*	Cellular carbon biomass formation: μ *M*	mol C cell^−1^ day^−1^
*b*	Carbon demand rate per cell: ρ + *q*	mol C day^−1^ cell^−1^
*Y*	Prokaryote carbon growth efficiency: (Δ*C*_r_ day^−1^) (Δ*C*_r_ day^−1^ + Δ*C*_b_ day^−1^)^−1^ or μ (μ + *r*)^−1^ or *q* (ρ + *q*)^−1^	unitless
*t*	time	day
*T*	Temperature	Celsius
*K*	Temperature	Kelvin
*R*	Molecular gas constant: 8.314	*J* (mol *K*)^−1^
*E*_a_	Activation energy in Arrhenius equation	k*J* mol^−1^

The symbol *Y* is used in reference to the classical microbiological literature (cf. Neidhardt et al., 1990). *Y* could otherwise be parameterized for example, in energy units (Heijnen, 1999) but in the ocean we lack the necessary information about the chemical nature of the organic substrates (Heijnen, 1999). If the extracellular organic carbon produced by microbes is neglected then the ecological role of microbes can be interpreted simply from the volumetric rates of growth (*C*_b_ day^−1^) and respiration (*C*_r_ day^−1^). However, in order to interpret *Y* as a physiological response of microbes to temperature, the specific respiration rate, *r* (*C*_r_ $C_{\text{b}}^{-1}$ *t*^−1^) and the specific growth rate, μ (*C*_b_ $C_{\text{b}}^{-1}$ *t*^−1^) need to be known (Cajal-Medrano and Maske, 1999), see Table 1 for symbols. *Y* can then be calculated:

(2) $$Y=\mu {\left(\mu +r\right)}^{-1}$$

If *r* would be proportional to μ then *Y* would be a constant, but this is not expected to be the case because respiration not only supplies the energy needed for biomass formation, but also for cell maintenance, movement and other activities that do not behave proportional to the specific growth rate (cf. Pirt, 1965; Heijnen, 1999). Ample literature has concerned itself with the non-growth related energy demand of cells mostly subsumed as maintenance energy. Little quantitative information is available about the maintenance energy of aquatic microbial communities. Generally an asymptotic increase in *Y* is expected with increasing μ independent of temperature (Cajal-Medrano and Maske, 1999). The Pirt model (1965) treats the metabolic rate control by energy nutrient limitation, invoking anabolic, and catabolic metabolisms to explain the increase in growth efficiency with faster growth rates. This directly addresses the question of carbon growth efficiency of microbes, but without considering the possibility that different activation energies for respiration and specific growth rates could imply temperature related changes in growth efficiency. Despite the long history of microbial research related to temperature, most of the published work is not applicable to microbial oceanography because the experimental work was performed with single species cultures, whereas natural microbial communities can be expected to adjust their phenotypic and genotypic profiles. Also, little experimental work so far was concerned with the temperature response of substrate limited marine microbes which is a growth condition typical of the ocean.

Here, we investigate the role of temperature in the relationship of *r* to μ and hence to *Y*. All experiments were done with an inoculum of natural microbial communities as we were considering the role of organotrophic microbial communities in the ocean rather than characterize specific strains. We discuss the data from 48 continuous seawater cultures of which 22 had been published earlier (Cajal-Medrano and Maske, 2005; Jiménez-Mercado et al., 2007). The previous publications focused on the control of growth efficiency by growth rate. Cajal-Medrano and Maske (2005) concluded that at a particular temperature and at lower growth rates, under substrate limitation the growth efficiencies are reduced as expected by the Pirt model (1965). Jiménez-Mercado et al. (2007) showed an increase in growth efficiency with higher temperature in unlimited turbidostats. In these cultures, the cell carbon and nitrogen increased with temperature and microscopically observed cell size was 3.4 times greater at 26°C than at 10°C. In this publication we are adding new data from 26 chemostats at different temperatures (Villegas-Mendoza, 2015) and are focusing on the different temperature response of the bacterial communities under limited (chemostats) and unlimited (turbidostat) growth conditions. Here we are showing a contrasting metabolic response of nutrient limited and—unlimited microbial cultures to temperature, information that should help to model the microbial response to temperature in the ocean.

## Materials and methods

### Cultures

Experimental methods have been described in detail in previous publications (Cajal-Medrano and Maske, 2005; Jiménez-Mercado et al., 2007). We ran temperature controlled, continuous cultures: 38 nutrient-limited chemostats were run between 14 and 26°C and specific growth rates (μ) between 0.05 and 6.0 day^−1^; 10 turbidostats with non-limiting nutrient supply were cultured between 10 and 26°C with μ ranging from 28 to 62 day^−1^. Turbidostat cultures are growing at the maximum growth rate defined by the temperature. The inoculum for the cultures was sampled in coastal waters close to Ensenada, Baja California (31.5° N, 116.5° W) or in the Gulf of California (31° N, 114.5° W) to investigate the potential importance of the origin of the inoculum on the results. The inoculum was prepared by filtering surface seawater samples through 0.8 μm polycarbonate filters (Whatman, Pleasanton, USA). Chemostat cultures were grown in 2 L and turbidostats in 1 L PTFE bottles to adjust to the rate of media consumption. Cultures were stirred with glass-encased magnets (140 rpm). Media was stored in 20 L polycarbonate containers. Apart from the media reservoir and the stirrers, all contact surfaces were either Teflon or silicon. All material in contact with cultures was previously sterilized. Pumping was by valve-less pumps (FMI) installed in the culture outflow to avoid contamination. Experimental methods have been described in detail in previous publications (Cajal-Medrano and Maske, 2005; Jiménez-Mercado et al., 2007).

Media was natural seawater aged for several months, ozonified and aged again; during the aging the media was filtered several times through GFF filters (Whatman, Buckinghamshire, England; 0.75 μm pore size). The aged seawater was supplemented with 20 or 100 μM glucose for the chemostats and turbidostats, respectively. Inorganic nutrients were added to the seawater as follows: (a) chemostats: 20 μmole L^−1^ NH_4_Cl, 5 μmole L^−1^ KH_3_PO_4_, 0.5 μmole L^−1^ FeCl_3_; b) turbidostats: 182 μmole L^−1^ NH_4_Cl, 43 μmole L^−1^ KH_3_PO_4_, 2 μmole L^−1^ FeCl_3_), and then acidified by percolating 450 mL min^−1^ CO_2_ for 5 min before autoclaving to avoid precipitation. After autoclaving the media was equilibrated by aeration with sterile air to return to saturated oxygen concentrations at room temperature. The sterility of the medium was regularly tested by passing a small volume of it into a ZoBell-enriched culture vessel.

The cultures were sampled at steady state when cell abundance stayed constant in chemostats for 2 days with steady state defined by less than 20% changes in cell abundance (Cajal-Medrano and Maske, 2005; Villegas-Mendoza, 2015); the steady state was reached after 3–19 days and there was no clear relation between this period and the dilution rate or the type of inoculum. In turbidostats the dilution rate was adjusted to maintain the cell abundance (*X*) constant. The steady state was defined by <10% change and it was reached after 10 h or more; the period was approximately inversely related to the maximum growth rate (Jiménez-Mercado et al., 2007). At steady state the specific growth, μ in chemostats and turbidostats was assumed to be equal to the dilution rate *D*, where *D* (d^−1^) = *F*/*V*, with *F* equal to the flow rate (L d^−1^) and *V* is the culture volume (L). In the case of turbidostats we adjusted the estimate of the growth rate taking into account the change in cell abundance over time prior to sampling, according to

(3) $$\mu =D+\ln(X_{1}X_{2}^{-1})\Delta t^{-1}$$

*X*_1_ and *X*_2_ are the microbial abundances at time 1 and 2 respectively; Δ*t* is the time interval in days between times 1 and 2.

We repeated some of the experiments using the same dilution rates and temperatures but with inoculum from the same site or from different locations but found no pattern (Cajal-Medrano and Maske, 2005; Jiménez-Mercado et al., 2007). The data of each repeat were included in our analysis as individual culture results.

### Microscope counts

Chemostat samples for epifluorescence microscope counts of microbes were collected daily from the culture outflow in scintillation vials and fixed with formaldehyde (2% final concentration). Turbidostat samples were collected several times per day. Samples were filtered on 0.2 μm black polycarbonate filters (Poretics) and stained with DAPI (Molecular Probes) (Turley and Hughes, 1992). Between 350 and 1,200 cells per sample were counted using wide-field epifluorescence microscopes with 100 x objectives. We observed no contamination of the cultures with protists.

### POC and PON

Particulate organic carbon and nitrogen samples from the culture suspension and the fresh medium were taken at the end of the experiments by filtering a volume of 300–400 mL using combusted (450°C, 2 h) glass fiber filters (GF/F, Whatman) with an effective pore size of 0.3 μm (Nayar and Chou, 2003) mounted in pre-combusted filter holders. Combusted GF/F filters are expected to retain all bacteria due to their reduced pore size after combustion (Nayar and Chou, 2003). The filtered samples and blank filters were lyophilized before analysis with a CHN analyser (Marine Science Institute, UCSB). We tried a second filter placed under the sample filter to correct for adsorbed organics (Maske and Garcia-Mendoza, 1994) but found that it did not significantly change the results. For some cultures two or three filter samples were taken and the results averaged. The prokaryote carbon biomass (*C*_b_) was calculated from the difference of particulate organic carbon (POC) in cultures minus POC in the media supplying the cultures.

### CO_2_ respired

CO_2_ respired was calculated as previously explained (Cajal-Medrano and Maske, 2005): in short, the culture or medium samples were titrated with HCl at 25°C using an automatic potentiometric technique in a closed titration cell (Hernández-Ayón et al., 1999; Dickson et al., 2007). A PC was used for controlling the syringe pump and storing the digitized pH data. Total CO_2_ (TCO_2_) was calculated directly from the derivative of the titration data, giving two inflection points from which total carbonate and alkalinity are computed. The precision of our titration is in the range of 0.15–0.4% (C.V.), equivalent to 3–8 μmol TCO_2_ L^−1^. The performance of our method was assessed by titrating seawater certified reference material (CMR). In average, we found a difference with the true value of TCO_2_ of 8.9 ± 1.5 μmol kg^−1^ (*n* = 3 samples of CMR). This variability is similar to that found by Camiro-Vargas et al. (2005) with samples of microalgae cultures and higher than Hernández-Ayón et al. (1999) with filtered seawater samples (8 μmol kg^−1^ for TCO_2_).

The difference of total CO_2_ between culture and media yielded *C*_r_. Assuming steady state the rate of CO_2_ produced is given by *D C*_r_ (μM CO_2_ day^−1^). The specific respiration rates were then calculated by dividing this rate by the steady-state biomass

(4) $$r=D\;C_{\text{r}}\;C_{\text{b}}^{-1}$$

We are implicitly assuming that the mean residence time of all elements in the cultures is *D*^−1^ (day).

### Determination of bacteria community composition

Samples for 16s rRNA gene (rDNA) amplicons were collected from last four cultures at steady state by filtering 250 mL of each culture. The total genomic DNA was extracted using the “Gentra Puregene Yeast/Bact Kit” according to the manufacturer's protocol (Qiagen, Valencia, CA, USA). Nucleic acids were sent to the Research and Testing Laboratory (Lubbock, TX, USA) for 454-pyrosequencing. Primers 28F (5′-GAG TTT GAT CNT GGC TCA G-3′) and 519R (5′GTN TTA CNG CGG CKG CTG-3′) were used for amplification of the variable regions V1-V3 of the bacterial 16S rRNA gene (La Duc et al., 2012). We did not attempt to detect the archaea community. Pyrosequencing reads were processed according to the protocol of the sequencing company (http://www.rtlgenomics.com/docs/Data_Analysis_Methodology.pdf), including de-multiplexing (SFF file generation), denoising, chimera detection, and taxonomic analysis using the NCBI database. The taxonomic levels used in the data analysis were based upon the following criteria; 97% identity (<3% divergence) were applied to resolve the species level, between 95 and 97% were used to define the genus level. The four sequences are published together with six more sequences in the NCBI database (https://www.ncbi.nlm.nih.gov/Traces/study/?acc=SRP099306) for public access purposes. The four sequences can be identified by the growth rate (μ: 0.2, 0.37, 0.8, 0.93 day^−1^) given in the data bank.

### Conversion factors for literature comparison

In the discussion below we compare our data with recent data reviews (Makarieva et al., 2008; DeLong et al., 2010). For this comparison, we used the conversion factors given in these publications to arrive at units used in our work: ratios of dry-weight to wet-weight = 0.3, carbon to dry-weight = 0.5, respiration coefficient (RQ) of 1.0, and 20 J (ml O_2_)^−1^.

### Calculations and statistics

To facilitate data comparison we provide *Q*_10_ values. The *Q*_10_ was calculated from the median temperature of the data set, because in the case that the data follow the Arrhenius model the *Q*_10_ values change for different temperature intervals.

All data were included in this data analysis without excluding outliers. Data from repeated experiments were treated the same as other experimental results. Type 2 regressions were calculated using the Matlab routine lsqfitma.m (http://www3.mbari.org/Products/Matlab_shell_scripts/regress/). Type 1 regressions were used when the abscissa was temperature.

## Results

### Microbial communities in continuous cultures

To show that the bacterial community in the chemostat cultures maintained species richness we estimated the microbial community based on pyrosequencing of 16S rDNA amplicons in the four most recent chemostat experiments (μ: 0.2, 0.37, 0.8, 0.93 day^−1^). The sequence data indicated that, when the cultures were terminated each culture community still included between 77 and 91 OTUs. In the four cultures the prevalent clades were *Roseobacter, Thalassospira xiamenensis, Rhodococcus, Sulfitobacter*, and *Marinobacter*.

### Specific respiration vs. growth rate

**Figure 1:**
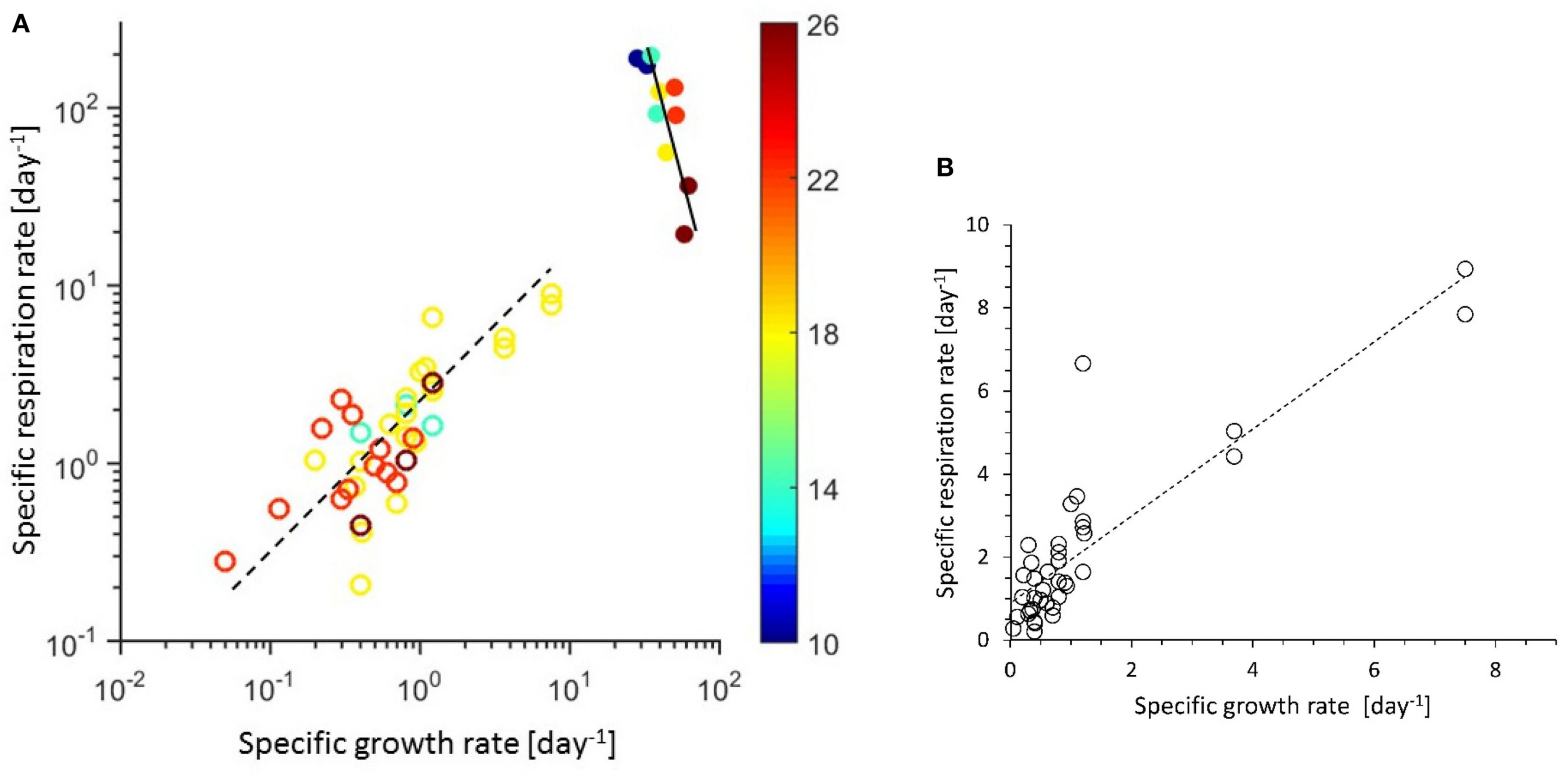
**(A)** Specific respiration rate (*r*, day^−1^) vs. specific growth rate (μ, day^−1^). Data points are colored according to the temperature scale, circles are chemostats, and filled circles are turbidostat data. Both axis are in log scale to increase data visibility. The broken line models the chemostat data (Equation 6, Table 2), the continuous line models the turbidostat data (Equation 7, Table 2). **(B)** Specific respiration rate (*r*, day^−1^) vs. specific growth rate (μ, day^−1^) of chemostat data plotted in linear scale (*r* = 1.05 μ + 0.089, *r*^2^ = 0.76).

The growth rate in nutrient-limited chemostats is controlled by the dilution rate, so no relationship of growth rate with temperature is expected, but the respiration rate could vary with temperature. In the turbidostat cultures the growth rate was controlled by temperature (Jiménez-Mercado et al., 2007) and the relationship (μ vs. *T*) is described by Equation 5 (Table 2). The relationships of specific respiration (*r*) and growth (μ) rate for all cultures are shown in Figure 1. The μ data of the chemostats (circles) range below 10 day^−1^ and turbidostat (filled circles) above 10 day^−1^. From Figure 1 it is obvious that the chemostat cultures behaved differently from the turbidostat cultures; chemostats showed a general increase in *r* with μ (Equation 6), whereas in turbidostats *r* decreased with higher μ (Equation 7). The data in Figure 1 are color coded for temperature and the growth rate increase with higher temperature in turbidostats (Equation 5) can be easily observed. Our experimental data (*T*, μ*, r*) are provided as supplemental data.

**Table 2:** Quantitative relationships.

	**Chemostat: Substrate limited**	**Turbidostat: Temperature limited**	**Q_10_**
μ vs. *T*	Not applicable	Equation 5: μ = e^∧^(m + n *T*) m = 2.984 ± 0.06 *n* = 0.0425 ± 0.0032 *r*^2^ : 0.96, *p* < 0.005	1.5
*r* vs. μ Figure 1	Equation 6: *r* = 10^∧^(m + n log(μ)) m = 0.3104 ± 0.0448 *n* = 0.8561 ± 0.1113 *r*^2^ : 0.61, *p* < 0.005	Equation 7: *r* = 10^∧^(m + n log(μ)) m = 7.666 ± 1.331 *n* = −3.509 ± 0.8161 *r*^2^ : 0.65, *p* < 0.005	
*r* vs. *T* Figure 2	No trend; *r*^2^ : −0.27, *p*: 0.11; median: 1.41 day^−1^	Equation 8: *r* = m + n *T* m = 262.5 ± 39.9 *n* = −8.479 ± 2.12 *r*^2^ = 0.67, *p* < 0.005	0.4
Y vs. *T* Figure 3	No trend; *r*^2^ : −0.05, *p*: 0.77; median: 0.34	Equation 9: ln(*Y*) = m + n *T* m = −2.827 ± 0.2996 *n* = 0.0889 ± 0.0159 *r*^2^ : 0.80, *p* < 0.005	2.6
*b* vs. 1,000/K Figure 4	No trend; *r*^2^: 0.25, *p*: 0.16; median: 10 fmol C(cell/day)^−1^	Equation 10: ln(*b*) = m + n 1000 K^−1^ m = 67.6 ± 5.49 *n* = −17.658 ± 1.596 *r*^2^ : 0.94, *p* < 0.005	8
*r* vs. *M*. Figure 5A	No trend for log-log data; *r*^2^ : < 0.01, *p* = 0.96	Equation 11: log(*r*) = m + n log(M) m = 2.315 ± 0.082 n = −0.443 ± 0.077 *r*^2^ = 0.77, *p* < 0.005	
*r* vs. *M* Figure 5A	Makarieva et al. (2008); weak trend, *r^2^* : 0.20, ρ : 0.0068		
ρ vs. *M* Figure 5B	Equation 12: log(ρ) = m + n log(*M*) m = 0.0996 ± 0.1070 *n* = 0.2520 ± 0.0370 *r*^2^ : 0.52, *p* < 0.005	Equation 13: log(ρ) = m + n log(*M*) m = 2.279 ± 0.081 *n* = 0.602 ± 0.077 *r*^2^ : 0.86, *p* < 0.005	
ρ vs. *M* Figure 5B	Makarieva et al. (2008), Equation 14: log(ρ) = m + n log(*M*)		
	m = −1.841 ± 0.138 *n* = 1.999 ± 0.133 *r*^2^ : 0.54, *p* < 0.005		
*b* vs. *M* Figure 6	Equation 15: log(*b*) = m + n log(*M*) m = 0.007 ± 0.182 *n* = 1.50 ± 0.22 *r*^2^ : 0.58, *p* < 0.005	Equation 16: log(*b*) = m + n log(*M*) m = 2.32 ± 0.043 *n* = 0.811 ± 0.045 *r*^2^ : 0.98, *p* < 0.005	
*b* vs. *M* Figure 6	DeLong et al. (2010), Equation 17: log(*b*) = m + n log(*M*)		
	m = 0.301 ± 0.179 *n* = 2.064 ± 0.203 *r*^2^ : 0.74, *p* < 0.005		

*For units see Table 1, Equations (Eq.) are listed in order of appearance in the text. See the Methods section for the calculation of regressions*.

### Specific growth, respiration rates, and *Y* vs. temperature

**Figure 2:**
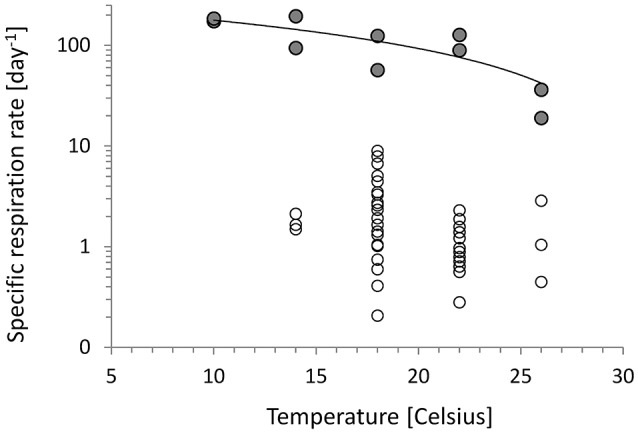
Specific respiration on log scale vs. temperature. Chemostats (circles) showed no statistical trend. Turbidostats (filled circles) showed decreasing *r* with temperature increase described by the continuous line (Equation 8, Table 2).

**Figure 3:**
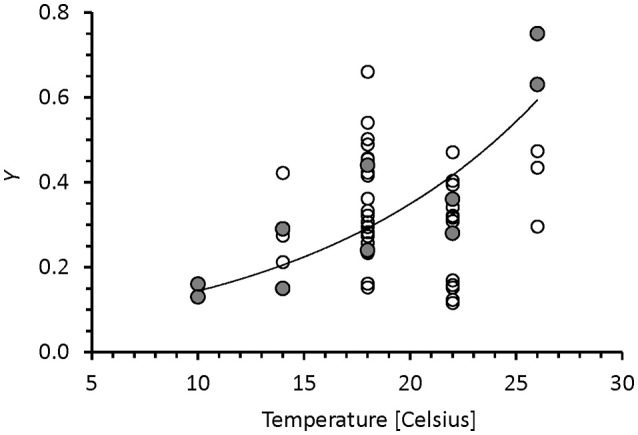
Prokaryote carbon growth efficiency vs. temperature. The chemostat data (circles) show no trend, but turbidostat data (filled circles) followed an exponential pattern depicted by the continuous line (Equation 9, Table 2).

In the chemostats, *r* showed no trend with temperature, but in the turbidostat cultures the specific respiration decreased with increasing temperature (Figure 2). The data could be described by a linear Type 1 regression, (Equation 8) and followed a Q_10_ of 0.4. The different patterns of chemostat and turbidostat *r* vs. μ in Figure 1 resulted in different patterns in growth efficiency (*Y*) with temperature. Figure 3 shows the relationship between *Y* and temperature; the *Y* of chemostats extended over the same data range as the turbidostat data, but showed no significant trend with temperature with an average value of *Y* = 0.34. The turbidostat data increased exponentially (Equation 9), corresponding to a *Q*_10_ of 2.6.

## Discussion

It is generally assumed that metabolic and growth rates follow empirically an Arrhenius type temperature dependence between the cardinal points (Neidhardt et al., 1990). Respiration rate is then expected to scale with

$$\ln(r)=\ln(I)+\left(-E_{\text{a}}{\left(RK\right)}^{-1}\right)$$

where *I* is the intercept, *R* the universal gas constant, *K* the temperature in Kelvin, and *E*_a_ is the activation energy that parameterizes the temperature response. Another parametrization to characterize the temperature response of microbes is the *Q*_10_. The *Q*_10_ assumes a simple exponential response different from the Arrhenius model, thus both parameterizations are not strictly compatible. The physiological response of microorganisms to temperature is more complex than suggested by the Arrhenius or *Q*_10_ parametrization and can affect specific metabolic pathways in different ways. To allow for a more detailed description of the metabolic response the concept of thermal performance curves (TPC) was recently introduced (Schulte, 2015). In general, TPCs are representations of one specific physiological property of single species. TPCs of one species may be dissimilar for different physiological activities as shown for phytoplankton (Baker et al., 2016). With respect to our work, the TPCs for *r* and μ of the different prokaryote strains within the community cannot be expected to follow the same pattern, but even single strain cultures may show significant differences in growth rate between individual cells (del Giorgio and Cole, 1998). In aquatic microbial communities the temperature response may be modified by the physiological interaction between members of the consortium, this includes cell lysis and converting part of the original substrate taken up by cells into chemically diverse organic cell debris that can then be recycled by other microbes. This interaction complicates the interpretation of bulk physiological properties of organotrophic communities. We provided glucose in the growth medium as the primary organic substrate, but given the trophic interaction in a microbial community, we need to assume that part of the community was nourished by recycled organics. Glucose was chosen as a substrate because it is metabolized by most microbes and glucose is found in the ocean (Rich et al., 1996; Skoog et al., 2002; Landa et al., 2014). One concern about the interpretation of steady state continuous cultures of natural bacterial communities is the selection of a few adapted species. We tried to keep culture turnover before steady state to a minimum and found in four chemostat cultures between 77 and 91 bacterial OTUs during steady state suggesting taxonomic variety that would allow for trophic interactions.

### Chemostat and turbidostat microbial communities show different temperature response

Figure 1 and Equation (6) (Table 2) show a clear difference in temperature response between nutrient-limited chemostat cultures and temperature limited turbidostat cultures. Because of the relative constancy of the *r* over μ ratio, the growth efficiency (*Y*) of chemostats showed no clear trend with growth rate and none with temperature (Figure 3). The pattern was very different from the turbidostat data in which the growth rate increased with temperature, but *r* decreased, resulting in an increase in *Y* with temperature (Equation 9). In Figure 3 the turbidostat data show a temperature dependence with a *Q*_10_ of 2.6. This value is very different from *Q*_10_ values reported for *Y* of microbes in a salt marsh estuary, ranging from −1.3 below 15°C to −3.6 between 15 and 30°C (Apple et al., 2006).

In a data review Yvon-Durocher et al. (2012) suggested a strong dependence of ocean ecosystem respiration with temperature. Rivkin and Legendre (2001) calculated a trend of decreasing *Y* with increasing temperature, using literature data of respiration spanning a wide range of latitudes based on *in situ* microbial production and DOC uptake or oxygen consumption in the dark of filtered (<1 μm) samples. They proposed that this trend may result from increasing respiration rates with temperature. White et al. (1991) showed a tendency of increasing growth rates with chlorophyll concentrations. Assuming that chlorophyll is inversely related to ocean temperature on global scales, then the trend observed by Rivkin and Legendre (2001) is perhaps a combined effect of temperature and substrate availability, as suggested by López-Urrutia and Morán (2007). Apple et al. (2006) also measured a change in the ratio of community respiration to microbial production with temperature in samples (<1 μm) taken in different parts of an estuary and during the annual cycle, resulting in a decrease of *Y* with temperature. Rivkin and Legendre (2001) and Apple et al. (2006) based their arguments on volumetric rates, because they lacked microbial biomass information; consequently their data do not lend themselves to physiological interpretations based on specific rates, as pointed out in relation to Equation (2).

### The carbon growth efficiency, *Y*

Turbidostat cultures yielded the highest values of *Y* near 0.8 (Figure 3) which are high compared to reviews of *Y* for planktonic bacteria (del Giorgio and Cole, 1998; López-Urrutia and Morán, 2007). Even the average value of *Y* = 0.34 of the chemostats (Figure 3) is higher than most oceanographic reports (López-Urrutia and Morán, 2007), but similar to mesocosm results (Dinasquet et al., 2013). In glucose-limited chemostat cultures (25°C, dilution rate 2.4 day^−1^) of a proteorhodopsin containing marine bacterium Courties et al. (2015) reported an average *Y* of 0.57; using Equation (2) we calculated their specific respiration rate to be 1.8 day^−1^ which would place their result close to our regression in Figure 1. We suggest that our high *Y* values are due to different aspects of our methodological approach: the use of glucose as an easily metabolized substrate; and the calculation of *Y* based on the measurement of respired CO_2_ and POC to define microbial biomass. The POC included not only living biomass but also detritus formed by the microbial community and retained by the sample filter. Because the central role of microbes in the ocean is the conversion of dissolved organics into particles that then enter the trophic web, the efficiency of the formation of all particulate organics from dissolved organics is relevant for ecological interpretations. We expected that the use of glucose as a substrate would support a high *Y* because glucose is easily assimilated, and because an organotrophic community that is mainly consuming a single type of substrate does not need to invest in diverse assimilation machinery. The relatively high *Y* might also be supported by the continuous cultures methodology which selects for the actively growing population and selects against slow growing or dormant cells. These latter cells would reduce community *Y* by adding to respiration without biomass formation.

### Cellular carbon demand rate

The temperature response of the cellular carbon demand rate of microbial communities can help in the interpretation of carbon cycling in the ocean. A better metric for the quantification of metabolic activity should be the cellular carbon demand rate (*b*) that includes the formation of carbon biomass and respired carbon, anabolism and catabolism. *b* is used here as a proxy for overall metabolic activity:

(5) $$b=\rho +q\;(\text{fmol C }(\text{cell }{\text{day}}^{-1})$$

**Figure 4:**
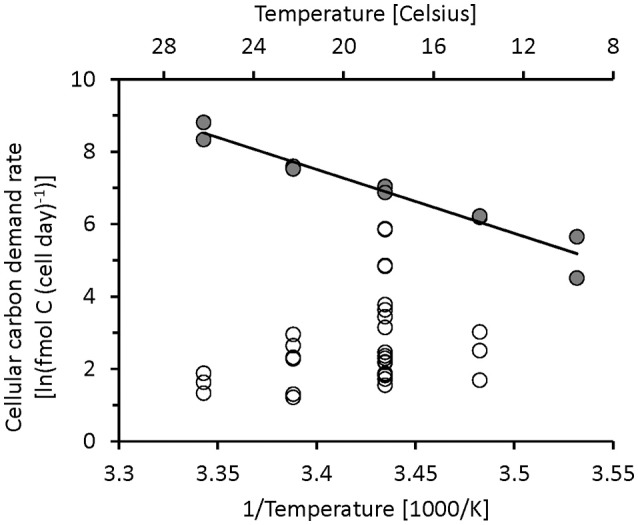
Natural log of the cellular carbon demand rate (fmol C (cell day)^−1^), calculated from the sum of specific growth rate and respiration, cell carbon and abundance vs. Kelvin^−1^. Chemostat cultures (circles) showed no change, but turbidostats (filled circles) showed an increase in carbon demand rate with higher temperature with *Q*_10_ of 8.1 as shown by continuous line (Equation 10, Table 2).

ρ = *rM* and *q* = μ*M* (Table 1). Figure 4 is cast in the linearized form of the Arrhenius plot, with the natural log of *b* plotted against the inverse of temperature. Chemostat data indicated no trend in Figure 4, but turbidostat data showed a strong increase in *b* with temperature (Equation 10). For the midpoint of the slope we calculated a *Q*_10_ of 8.1. This *Q*_10_ was higher than values for physiological rates found in the literature, for example *Q*_10_ values between −1 and 4.4 were reported for different physiological rates of microbes in a salt marsh estuary (Apple et al., 2006).

### Cellular carbon vs. metabolic rates

Allometry scales the metabolic activity (*MA*) of an organism to its biomass (*M*), for example: *MA* = *M*^∧^*Exp*. The exponent (*Exp*) defines the allometric properties of groups of organisms and can be included in numeric ecosystem models. Historically *Exp* was expected to be <1.0, thus the specific metabolic activity of an organism decreased with its biomass. Makarieva et al. (2005, 2008) argued that the ratio of metabolic activity to biomass was similar across the full organismal size range of the different groups, but DeLong et al. (2010) showed that different groups of organisms had specific allometric exponents. For prokaryotes they found *Exp* ~ 2.0. In their reviews Makarieva et al. (2005, 2008) and DeLong et al. (2010) used published respiration rates from monospecific prokaryote cultures as a metric for metabolic activity after converting respiration rate to power (Watt). They used wet weight for the size of the organism for easier comparison between different groups of organisms. Both publications adjusted metabolic rates for easier comparison to 20 or 25°C using a Q_10_ = 2.0. For a comparison of our data with their prokaryote data sets we converted their values to cellular respiration (ρ, mol C cell^−1^ day^−1^), cellular carbon (*M*, mol carbon cell^−1^), and adjusted the rates to the original experimental temperatures using the same conversion factors as in the reviews.

**Figure 5:**
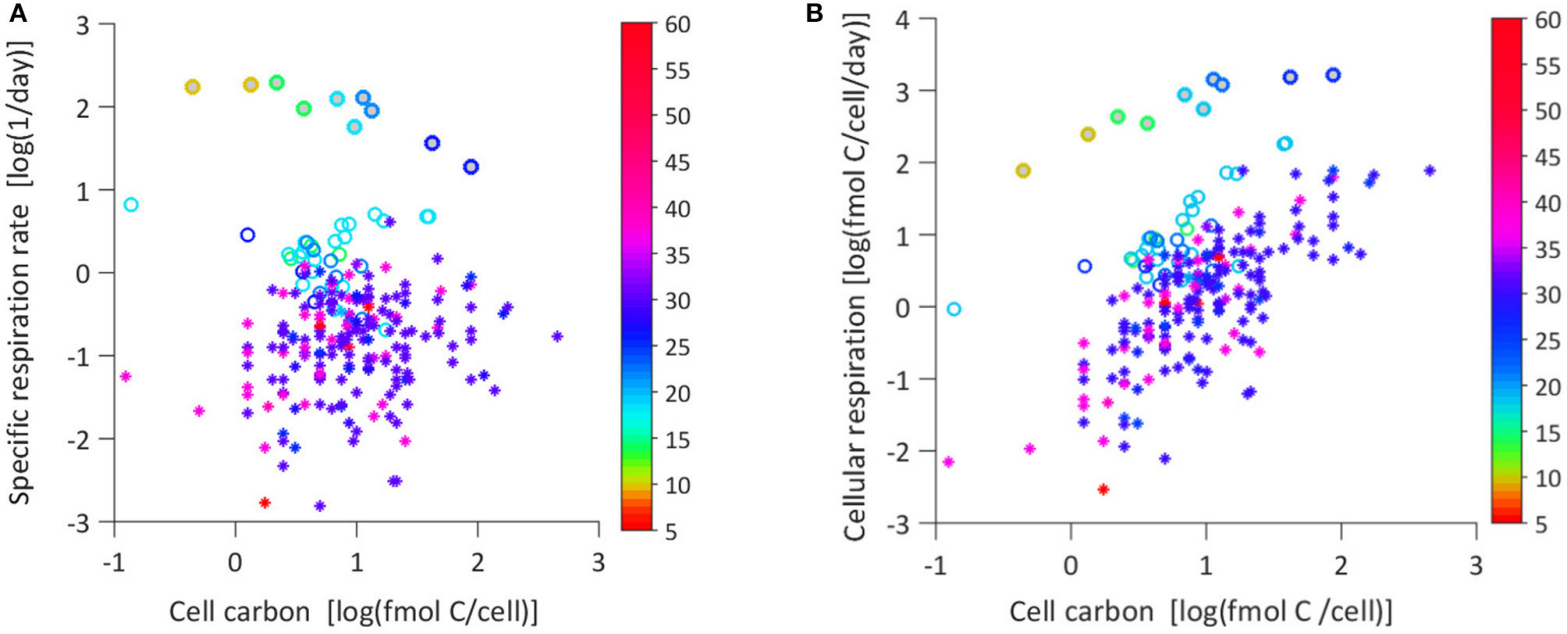
**(A)** Specific respiration vs. cell carbon, color coded by temperature. Chemostats (circles), turbidostats (circles filled gray, Equation 11, Table 2); the data in Makarieva et al. (2008) (stars) were recalculated to the original experimental values. **(B)** The same data as in figure **(A)** presented here as cellular respiration rate vs. cellular carbon, and color coded for temperature: Chemostats (circles, Equation 12, Table 2), turbidostats (circles filled gray, Equation 13), Makarieva et al. (2008) (stars, Equation 14).

In Figure 5A we compare the cell carbon (*M*) to specific respiration rate (*r*); in turbidostat cultures *r* decreases with carbon-rich cells and shows a clear trend with temperature indicated by symbol color. The chemostat *r* partially overlaps with the data from Makarieva et al. (2008), but our *r* values are significantly higher than the values listed by Makarieva et al. (2008), median: 0.162 day^−1^. Neither data set shows a trend in *r/M* space or with temperature. In Figure 5B we show the same data as in Figure 5A but converted to cellular respiration rate (mol C cell^−1^ day^−1^). All data show significant relations with cellular carbon (*M*). The data of Makarieva et al. are lower than our chemostat data. The Makarieva et al. data show the steepest data slope (Equation 14), a lower slope for chemostats (Equation 12) and the lowest for turbidostats (Equation 13). The coloring of the data points only indicates a trend with temperature for the turbidostat data.

**Figure 6:**
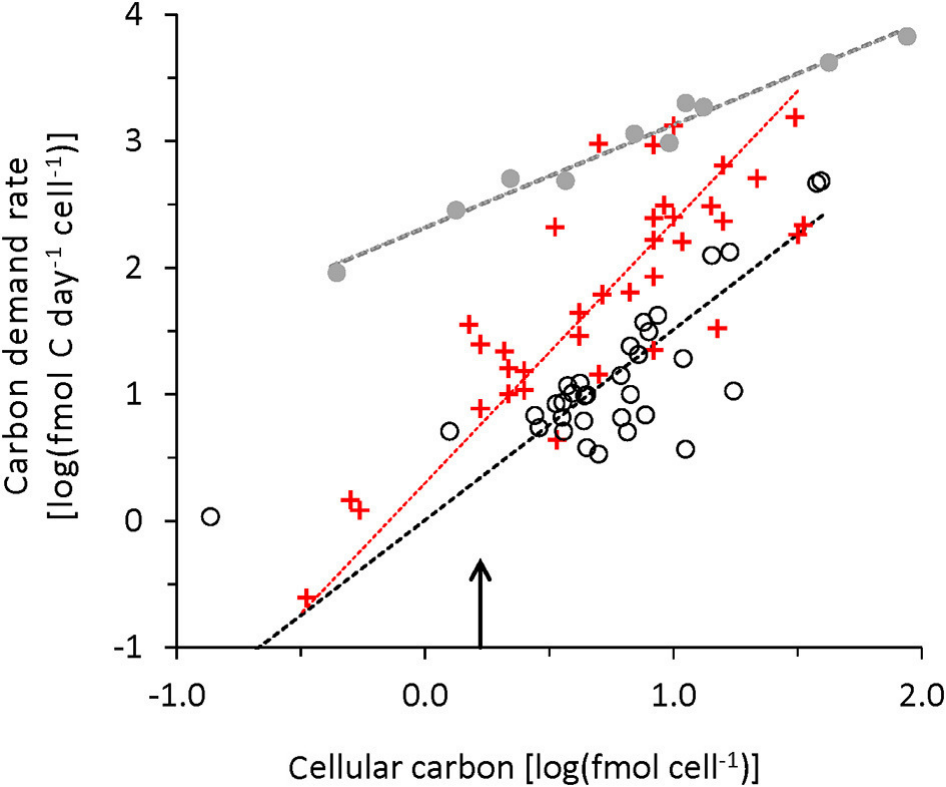
Cellular carbon demand rate vs. decadal log of cellular carbon: Our data at experimental temperature (chemostats, black circles and broken black line, Equation 15, Table 2); turbidostats, gray dots, and broken gray line (Equation 16). DeLong et al. (2010) at 20°C (red crosses and red line Equation 17). The arrow indicates the cellular biomass of 20 fg C/cell frequently used in oceanographic literature as a reference value.

DeLong et al. (2010) and Makarieva et al. (2008) used respiration rate as a metric for metabolic activity but the respiration rate is considering only the catabolic part of metabolic activity and neglects the anabolic part (Figure 5). Data provided by DeLong et al. (2010) allowed to calculate *b* but only for 20°C, so in Figure 6 we compare their *b* with the values from chemostat and turbidostat at experimental temperature. Data from DeLong et al. (2010) show cellular carbon demand rates between our chemostat and turbidostat data. Turbidostat data show a strong relationship (Equation 16), but DeLong et al. (Equation 17) and the chemostat data (Equation 15) are noisier. The slope for DeLong et al. (2010) is significantly steeper than for our data, implying that the specific carbon demand rate, *b* increases more strongly with bigger cells. We do not have the data to evaluate if this behavior is related to the adjustment to 20°C of the data in DeLong et al. (2010).

The comparison of our data with the literature data yields a similar cellular carbon range but different patterns of respiration, growth and carbon demand rate. Our chemostat metabolic rates are higher than the data listed by Makarieva et al. (2008). This might be related to the much higher biomass densities of the cultures in the cited literature, which might affect the physiological state of the culture. Turbidostat data always show the highest metabolic rates and a data pattern that is statistically better defined, but with a different trend from chemostat and literature data. The chemostat data always show lower correlation coefficients than either turbidostat or the literature data. The relatively well-defined statistical trend in the literature data is surprising considering that a wide range of different methods was used for cultivation, to measure either respiration or cell size. We propose that one reason why the literature data have relatively high correlation coefficients is that they are based on mono-specific cultures, whereas we used bacterial communities in our continuous cultures. The turbidostat cultures probably behaved more like the mono-specific cultures because the strong selection for nutrient-replete, temperature-controlled growth forced a strong selection minimizing the community interaction.

When comparing our culture results with oceanographic data it should be considered that oceanographic research is still struggling with the measurement of basic metabolic rates. We still lack sufficiently sensitive and generally accepted methods that allow the determination of microbial respiration using short incubation times without pre-incubation size filtration, methods that would minimize physiological changes during incubation (Pomeroy et al., 1994) and changes in taxonomic composition (Gattuso et al., 2002). Methodological differences such as potential overestimation of microbial oxygen respiration rate measurement using the Winkler technique in the field (Apple et al., 2006; Wong and Li, 2009) and our use of CO_2_ determination under steady state conditions could partly be responsible for the difference in average *Y* found in our chemostat data and the lower average growth efficiency reported in the oceanographic literature. Another possible explanation might be the documented, but as yet unexplained variation in respiratory quotient (Berggren et al., 2012; Romero-Kutzner et al., 2015). Our main conclusion is that the specific respiration of prokaryote communities under substrate limited growth condition shows no trend with temperature in the range of temperature tested in our study. The carbon demand rate in chemostat cultures (Figure 4) or the specific or cellular respiration rate in mono-specific culture data from the literature, or in our chemostat data, did not show patterns related to temperature or cell carbon (Figures 5A,B). In contrast, current ecological modeling approaches consider adjustments of metabolic rates based on temperature and on the biomass of the organism using, for example the metabolic theory in Ecology (MTE; Brown et al., 2004; Allen et al., 2005). The temperature adjustment in MTE is based on Arrhenius-type temperature adjustment, but other models use Q_10_ as an alternative scaling of metabolic activity to temperature. The concept of temperature adjustment of metabolic rate is based on cultures growing at temperature limited rates, which is probably a rare occurrence in aquatic environments. For the ocean the respiration rates of bacteria communities currently cannot be adjusted with temperature response functions.

## Author contributions

HM, RC-M, and JV-M: Measurements, concept, and writing.

### Conflict of interest statement

The authors declare that the research was conducted in the absence of any commercial or financial relationships that could be construed as a potential conflict of interest.
